# Non-stop management of an electroconvulsive therapy unit (ECT-U) during the first two months of COVID-19 lockdown in Spain

**DOI:** 10.1192/j.eurpsy.2021.2069

**Published:** 2021-08-13

**Authors:** V. Llorca-Bofí, I. Batalla, M. Adrados-Pérez, E. Buil-Reiné, J. Pifarré, A. Torrent

**Affiliations:** Psychiatry Department, HU Santa Maria, Lleida, Spain

**Keywords:** Electroconvulsive therapy, COVID-19, lockdown

## Abstract

**Introduction:**

Since the declaration of the national lockdown in Spain on March 14th until the publication of the SEPB recommendations on May 11th, most of the ECT-U closed or drastically reduced their activity.

**Objectives:**

To present our non-stop management of an ECT-U during the first two months of COVID-19 lockdown in Spain.

**Methods:**

We retrospectively analysed the time between sessions, the clinical, pharmacological and electrical data records of maintenance patients (m-ECT) and compared them with their own records in the two-month period prior to COVID-19. We analysed the length of admission, clinical, pharmacological and electrical records in hospitalized patients (i-ETC) and compared them with patients from the entire year prior to COVID-19 paired by age, sex and diagnosis.

**Results:**

The ECT-m programme included 17 patients: we postponed the ECT in 8 patients; 1 patient was hospitalized and 8 patients continued normally. The time between m-ECT increased by 8.37±4.89 days (p=0.018) without relapse. During the COVID-19 period, we performed ECT-i in 14 patients without new infections. In i-ECT the duration of admissions increased by 22.1 ±1.2 days (p=0.006), the load increased by 81.53 ±87.8 mC (p=0.027) and the time of the electrical seizure decreased by 7.9 ±9.2 seconds (p=0.037).

**Conclusions:**

The modifications that reach a statistical significance are explained by the readjustment of the ECT-U, with no clinical significance. With the appropriate measures, neither m-ECT nor i-ECT were discontinued. Thus we maintained adequate patient management.

**Disclosure:**

No significant relationships.

